# Connective Tissue Growth Factor (CTGF/CCN2) enhances lactogenic differentiation of mammary epithelial cells via integrin-mediated cell adhesion

**DOI:** 10.1186/1471-2121-11-35

**Published:** 2010-05-24

**Authors:** Bethanie L Morrison, Cynthia C Jose, Mary Lou Cutler

**Affiliations:** 1 Department of Pathology, F. Edward Hebert School of Medicine, Uniformed Services University of the Health Sciences, Bethesda, MD 20814, USA; 2 Current Address: Drug Mechanism Group - Developmental Therapeutics Program, SAIC Frederick/NCI-FCRDC, Bldg. 320, Rm 8, Frederick, MD 21702, USA

## Abstract

**Background:**

Connective Tissue Growth Factor (CTGF/CCN2), a known matrix-associated protein, is required for the lactogenic differentiation of mouse mammary epithelial cells. An HC11 mammary epithelial cell line expressing CTGF/CCN2 was constructed to dissect the cellular responses to CTGF/CCN2 that contribute to this differentiation program.

**Results:**

Tetracycline-regulated expression of CTGF/CCN2 in HC11 cells enhanced multiple markers of lactogenic differentiation including β-casein transcription and mammosphere formation. In a separate measure of mammary differentiation the addition of CTGF/CCN2 to cultures of MCF10A cells increased the development of acini *in vitro*. In HC11 cells the elevated levels of CTGF/CCN2 diminished the requirement for extracellular matrix proteins in the activation of β-casein transcription, indicating that CTGF/CCN2 contributed to lactogenic differentiation through the regulation of matrix dependent cell adhesion. CTGF/CCN2 expression in HC11 cells increased expression of extracellular matrix proteins and integrins, enhanced the formation of focal adhesion complexes, and increased survival signaling. In addition, HC11 cells adhered to immobilized CTGF/CCN2 and this was inhibited by function-blocking antibodies to the integrins α6 and β1, and to a lesser degree by antibody to β3 integrin.

**Conclusions:**

CTGF/CCN2 expression in HC11 cells led to an increase in multiple markers of lactogenic differentiation. The mechanisms by which CTGF/CCN2 contributed to lactogenic differentiation include direct binding of CTGF/CCN2 to integrin complexes and CTGF/CCN2-induced matrix protein expression resulting in elevated integrin functionality.

## Background

The development of the mammary gland is hormonally regulated [1], but the actions of locally-derived growth factors and the interaction of mammary epithelial cells with their surrounding stroma are also critical factors for successful development [2]. Mammary epithelial cells interact with the extracellular matrix predominantly through the stromal components collagen and laminin [3-5]. Lactogenic differentiation is associated with the deposition of laminin-rich matrix by the epithelial cells [6,7] and the degree of differentiation of mammary epithelial cells correlates with their response to basement membrane and stromal protein-induced signals. In addition, the production of milk proteins by the secretory epithelium is dependent on the presence of specific mitogens [8-10], cell-cell contact [11,12], stimulation by the lactogenic hormone prolactin [13-15], and interaction with the extracellular matrix [7,16-18].

β1 integrin expression is required for the survival of epithelial cells during differentiation [19] and it contributes to mammary gland development and morphogenesis [20,21]. The interaction of β1 integrin with laminin is critical for the initiation of the transcription of the milk protein β-casein [22,23]. In addition, during lactogenic differentiation the activation of the prolactin receptor ultimately results in the translocation of phosphorylated Stat5 dimers to the nucleus where they bind DNA and regulate transcription [13,14,24], and integrin-mediated adhesion is critical for the activation of Stat5 [25]. *In vitro *studies of the interaction between mammary epithelial cells and basement membrane proteins during transcription of milk proteins recently implicated the *SWI/SNF *transcription factor, Brg1, in translating signals from the stroma to the activation of the β-casein promoter [26].

Our previous work determined that Connective Tissue Growth Factor (CTGF/CCN2), a known stromal mediator, is highly up-regulated during the lactogenic differentiation of mouse mammary epithelial cells in a glucocorticoid-dependent response [27,28]. That study demonstrated that transient expression of CTGF/CCN2 enhanced β-casein transcription during the lactogenic differentiation of mouse mammary epithelial cells and that siRNA-mediated depletion of CTGF/CCN2 blocked the process [27]. CTGF/CCN2 is a member of the CCN family of matrix-associated proteins, which are known to be involved in processes including the regulation of growth, differentiation, migration and adhesion [29,30]. Members of the CCN family are comprised of 4 homology domains: the N-terminal insulin-like growth factor binding protein (IGFBP1) homology domain, followed by the von Willebrand C (VWC) repeat domain, the thrombospondin type 1 (TSP1) repeat domain, and the C-terminal cysteine knot (CT) domain [31]. CTGF/CCN2 is known to interact with β1 integrin complexes through its TSP1 and C-terminal domains [32,33]. Because functional β1 integrin complexes are required for lactogenesis *in vivo *and *in vitro*, our studies focused on the effect of CTGF/CCN2 expression on this axis in mammary epithelial cells.

The studies presented here employed HC11 mouse mammary epithelial cells, a cell line capable of lactogenic differentiation *in vitro *[34-36], that has been used in our previous studies [27,37,38]. HC11 cells are non-transformed, immortalized, and undergo lactogenic differentiation upon stimulation with dexamethasone, insulin and prolactin [34,39]. To address the mechanism by which CTGF/CCN2 contributes to the regulation of lactogenic differentiation, CTGF/CCN2 was expressed under the control of a Tetracycline-regulated promoter in HC11 cells. The results confirm and extend our previous findings that CTGF/CCN2 mediates the enhancement of multiple markers of lactogenic differentiation in HC11 cells [27]. In addition, ectopic expression of CTGF/CCN2 increased the formation of focal adhesion complexes, integrin-mediated survival signaling and cell adhesion. Hence, these findings suggest that CTGF/CCN2 acts to stabilize the cell-matrix interactions required for cell survival by multiple mechanisms, and this translates directly and indirectly into enhanced lactogenic differentiation as measured by the subsequent phenotypic changes and the transcription of β-casein.

## Results

### Ectopic CTGF/CCN2 expression enhanced the lactogenic differentiation of HC11 cells

Previous studies conducted in our laboratory demonstrated that CTGF/CCN2 was transcriptionally regulated by the glucorticoid dexamethasone during the induction of lactogenic differentiation in HC11 cells, and that elevated CTGF/CCN2 expression was required for the differentiation program [27]. To elucidate the mechanism by which CTGF/CCN2 enhances the lactogenic differentiation of mammary epithelial cells, a stable HC11-based cell line was created by employing inducible expression of CTGF/CCN2 under the control of a Tet-responsive promoter (TRE). Analysis of these cells revealed that the expression of vector-encoded CTGF/CCN2 readily increased following the removal of doxycycline from the culture media [27]. Following induction of CTGF/CCN2 expression in HC11 cells its effect on lactogenic differentiation was evaluated by the addition of the lactogenic hormone mixture of dexamethasone, insulin, and prolactin (DIP) to the cultures. Known markers of lactogenic differentiation, including β-casein transcription and mammosphere formation, were analyzed.

**Figure 1:**
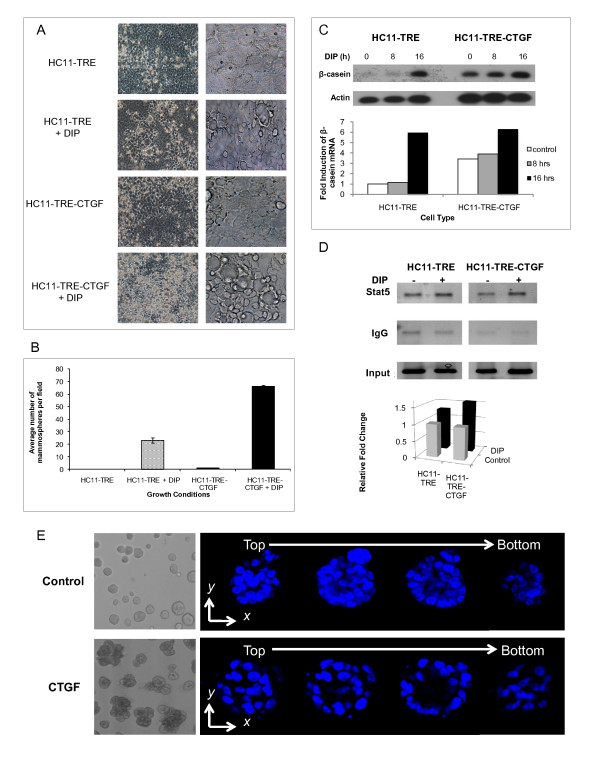
**Ectopic CTGF/CCN2 expression in HC11 cells enhances lactogenic differentiation**. (**A**) HC11-TRE and HC11-TRE-CTGF cells were grown to confluence and exposed to DIP. The cells were photographed at 120 hrs post-DIP addition and are shown at 20× and 40× magnification. The images shown representative of five fields. Mammospheres are bubble-like structures. (**B**) Mean is the result of the average number of mammospheres in five low power microscopic fields. Data is expressed as the mean + S.D. (**C**) HC11-TRE and HC11-TRE-CTGF cells were stimulated with DIP for 0, 8, or 16 hours. RNA was extracted and examined by Southern blotting. For quantitation, expression levels of β-casein were normalized to actin and expressed as a fold induction of β-casein mRNA compared to the HC11-TRE control. (**D**) Chromatin immunoprecipitation analysis of β-casein promoter. HC11-TRE and HC11-TRE-CTGF cells were grown in serum-free media for 24 hours prior to being induced with DIP for 16 hours. (Top) PCR analysis of DNA immunoprecipitated with either an anti-Stat5 antibody or control IgG using primers to amplify the β-casein promotor. (Bottom) Graphical analysis of PCR results from DNA immunoprecipitated with an anti-Stat5 antibody, normalized to the amount of input DNA. Light columns = no DIP, dark columns = DIP (**E**) CTGF/CCN2 enhances the number and formation of MCF-10A acini in Matrigel. MCF-10A cells were suspended in Matrigel and seeded on Matrigel in 8 well chamber slides. Cells were grown in the absence or presence of CTGF/CCN2 (50 ng/ml) for 20 days. Images in depict acinar structures in culture by phase contrast microscopy at magnificantion 10×. The cells were fixed and the nuclei stained with DAPI. Shown are serial confocal cross sections of acinar structures with respect to the Z axis. The sequential sections reveal the hollow lumen (magnification 20×).

The formation of domed structures called mammospheres occurs during lactogenic differentiation of HC11 cells in culture and serves as a phenotypic marker of this process [40,41]. The effect of CTGF/CCN2 expression on mammosphere formation was examined following stimulation of HC11-TRE-CTGF and the vector control HC11-TRE cells with DIP for five days. The HC11-TRE-CTGF cells displayed enhanced mammosphere formation, both in the size (Figure 1A) and number (Figure 1B), compared to the identically treated vector control cells.

The transcription of the milk protein β-casein is a marker of lactogenic differentiation. Our previous studies demonstrated that transient expression of CTGF in HC11 cells enhanced β-casein transcription compared to control cells [27]. Hence, confluent cultures of HC11-TRE-CTGF and HC11-TRE cells were stimulated with lactogenic hormone and assayed for differentiation by following β-casein transcription. Because endogenous CTGF/CCN2 is rapidly induced by dexamethasone, a component of the lactogenic hormone mix, the expression of β-casein RNA was examined in untreated cells and prior to 24 hours of DIP treatment. At 0 and 8 hours post DIP stimulation the effect of vector-encoded CTGF/CCN2 on this process (Figure 1C) was detected. The results indicated a higher level of β-casein transcription at 8 hours in the HC11-TRE-CTGF cells compared to the HC11-TRE cells, confirming that CTGF/CCN2 expression enhances transcription of β-casein in the HC11 cell line. In addition, the Tet-regulated CTGF/CCN2 expression resulted in low but detectable β-casein in the absence of added lactogens (T = 0) suggesting that a low level of lactogenic hormone, presumably contributed by serum in the media, could induce β-casein transcription in the presence of CTGF/CCN2.

The contribution of CTGF/CCN2 to β-casein promoter activation was assessed by evaluating the binding of Stat5 to the β-casein promoter via a chromatin immunoprecipitation assay (ChIP). Stimulation of HC11 cells with prolactin activates Jak2 resulting in Stat5 phosphorylation, dimerization, nuclear translocation and DNA binding. Control and CTGF-expressing HC11 cells were stimulated with the lactogenic hormone mix for 24 hours. The treated cells were fixed in formaldehyde, the nuclei were isolated and sonicated, and Stat5-DNA complexes were recovered by immunoprecipitation. The β-casein proximal promoter was amplified from the purified recovered DNA in order to determine the level of Stat5 DNA binding. Results, shown in Figure 1D, revealed that elevated CTGF/CCN2 expression led to a modest but reproducible increase in the binding of Stat5 on the β-casein proximal promoter in the induced but not the unstimulated cells. In repeated experiments (N = 5) the outcome ranged from 1.25-2.1 fold greater binding in TRE-CTGF than the TRE control cells. The fact that detection of Stat5 promoter binding required extended DIP-stimulation (i.e. 24 hours) diminished the differences between the control and CTGF/CCN2-expressing cells that were observed at the level of β-casein RNA. However, this result supported the finding of increased β-casein transcription in CTGF/CCN2-expressing cells.

### CTGF/CCN2 increased acinar formation by MCF10A cells

The formation of polarized acini-like spheroids that mimic aspects of the architecture of the mammary gland *in vivo *is a marker of differentiation for the immortalized human mammary epithelial cell line MCF10A [42]. The MCF10A cells form spheroids on Matrigel and this is followed by apoptosis-driven clearing of the lumen of these structures. The formation and differentiation of the spheroids relies on integrin engagement for the orientation and polarization of the cells to form three dimensional structures. Because CTGF/CCN2 expression enhanced the formation of the HC11 cell domed mammospheres, the effect of CTGF/CCN2 on the formation of the acinar structures by MCF10A cells was examined. CTGF/CCN2 was supplied to the MCF10A cells exogenously in a purified and soluble form. In this assay MCF10A cells were suspended in Matrigel in the presence or absence of CTGF/CCN2 and overlayed onto a solidified layer of Matrigel. The cultures were maintained for 20 days, at which time the cells were fixed, stained and analyzed by confocal microscopy. The images, shown in Figure 1E, depict individual DAPI-stained acinar structures in the presence or absence of CTGF/CCN2 as sections from top to bottom of the acinus. Initially, the presence of CTGF/CCN2 in the media enhanced both the size and the number of the spheroid structures formed by the MCF10A cells. However, over time the MCF10A cells cultured in the presence of CTGF/CCN2 also exhibited a high level of differentiation. This was characterized by a structured epithelial cell layer surrounding a hollow lumen. Thus, CTGF/CCN2 influenced the growth and differentiation of both HC11 mouse mammary epithelial cells in two-dimensional culture as well as MCF10A mammary epithelial cells in three-dimensional culture.

### CTGF/CCN2 functioned via matrix interaction during β-casein transcription in HC11 cells

**Figure 2:**
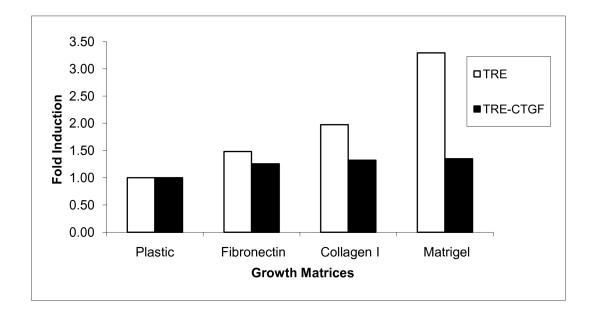
**CTGF/CCN2 contributes to the epithelial-matrix interaction during lactogenic differentiation in HC11 cells**. HC11-TRE and HC11-TRE-CTGF cells were seeded in serum-free conditions on respective coated or uncoated tissue culture dishes and once confluent, induced to differentiate with DIP for 48 hours. Total RNA was harvested and levels of β-casein transcript were determined by northern blot analysis and normalized to actin. Contribution of the matrix proteins to β-casein transcription can be seen by fold induction analysis of the RNA (white bars = HC11-TRE, dark bars = HC11-TRE-CTGF).

Previous studies have shown that CTGF/CCN2 acts as a stromal mediator by binding to integrins and other components of the extracellular matrix [43,44], and the activation of β1 integrin complexes by matrix components is required for β-casein transcription and lactogenic differentiation [45]. Therefore, the effect of CTGF/CCN2 on extracellular matrix regulation of β-casein transcription was examined. HC11-TRE-CTGF and vector control cells were seeded on fibronectin-, collagen I-, or Matrigel-coated or uncoated tissue culture dishes. Following stimulation of the cells with lactogenic hormone, RNA was extracted and used to analyze β-casein expression by northern blot. Results are shown as the fold induction of β-casein transcription in each cell line grown on matrix proteins compared to the level of transcription on plastic (Figure 2). The results demonstrated a greater than 3-fold increase in β-casein transcription in HC11-TRE vector control cells exposed to extracellular matrix components compared to tissue culture plastic. In contrast, HC11-TRE-CTGF cells did not display a significant increase of β-casein transcription when grown on fibronectin, collagen I or Matrigel. Thus, it appears that ectopic expression of CTGF/CCN2 partially abrogates the influence of matrix components required to initiate β-casein transcription. Moreover, the results suggest that CTGF/CCN2 contributes significantly to the collagen and laminin receptor engagement which is required for the initiation of HC11 mammary epithelial cell differentiation and β-casein transcription.

### CTGF/CCN2 stimulation contributed to HC11 cell proliferation and survival

**Figure 3:**
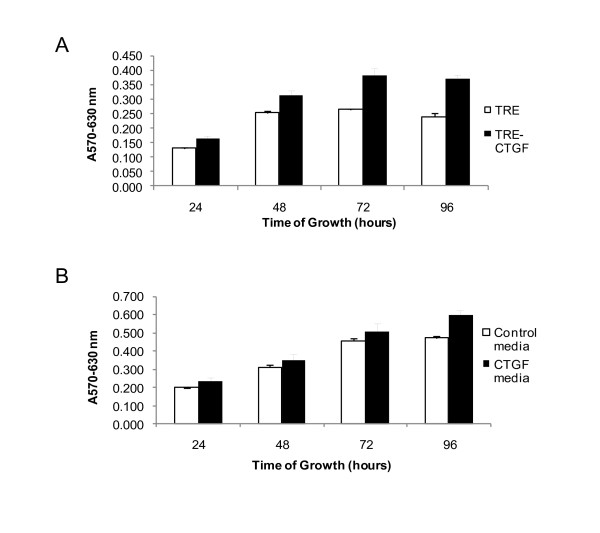
**CTGF/CCN2 stimulation contributes to HC11 cell proliferation**. (**A**) HC11-TRE and HC11-TRE-CTGF cells were seeded in 96-well microtiter plates in serum-free media with EGF (10 ng/ml). (**B**) HC11 cells were seeded in serum-free media with EGF (10 ng/ml) plus serum-free conditioned media from HC11-TRE or HC11-TRE-CTGF (1:1 ratio). Cell proliferation was determined at 24, 48, 72, and 96 hours post addition of EGF using the MTT assay. The results are reported as the mean + S.D. of four determinations. *, p < 0.005.

CTGF/CCN2 increased mitogenesis in numerous types of cells. Previously published data from our lab demonstrated that elevated expression of CTGF/CCN2 in HC11 cells resulted in enhanced expression of β1 integrin and the activation of focal adhesion kinase (FAK) [27]. Thus, to determine the contribution of CTGF/CCN2 to integrin-mediated processes that effect the growth of HC11 cells, CTGF/CCN2-expressing HC11 cells were compared to vector control cells for proliferative capacity in the absence of serum. The proliferation was determined by MTT assay (Figure 3A) and the results indicated HC11-TRE-CTGF cells displayed significantly more proliferative capacity than the HC11-TRE vector control cells. In particular, proliferation was sustained by CTGF/CCN2 expression at later times when the HC11 vector control cells ceased proliferation.

CTGF/CCN2 is a secreted protein and approximately half of the CTGF produced by the HC11-TRE-CTGF cells is found in the media [27]. Therefore, conditioned media from HC11-TRE-CTGF cells was harvested and also used to determine the effect of CTGF/CCN2 on the growth of HC11 cells. Results, shown in Figure 3B, indicated that CTGF/CCN2-conditioned media sustained the growth of HC11 cells longer than conditioned media from HC11-TRE cells. Again, the most significant differences were observed at later time points. Together with the effect of endogenously produced CTGF/CCN2, these results suggest that CTGF/CCN2 played a role in the proliferation of cells especially in the absence of serum. However, neither HC11 vector control, nor HC11 cells expressing or treated with CTGF/CCN2, proliferated in the absence of EGF (data not shown). This indicated that the effect(s) of CTGF/CCN2 in the HC11 cells differed from those of EGF on the same cells.

**Figure 4:**
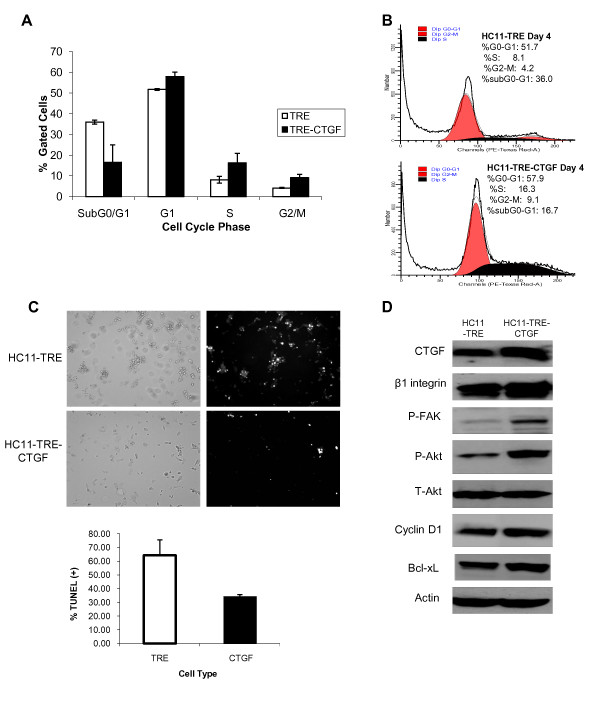
**CTGF/CCN2 sustains the survival of mammary epithelial cells**. (**A**) HC11-TRE and HC11-TRE-CTGF cells were seeded in serum-free media in the presence of EGF (10 ng/ml) and harvested after 96 hours. Cells were fixed in ethanol and stained with propidium iodide (50 μg/ml) prior to cell cycle analysis by flow cytometry. Data is representative of three determinations. Percentage of total gated cells in each phase of the cell cycle is shown. (**B**) Graphical analysis of cell cycle distribution by DNA content in HC11-TRE cells or in HC11-TRE-CTGF cells. (**C**) HC11-TRE and HC11-TRE-CTGF cells were seeded on coverslips in serum-free media in the presence of EGF (10 ng/ml) for 96 hours, then fixed and apoptosis was detected by TUNEL. Left panels depict cell morphology, while the panels on the right display positive TUNEL staining in white. Quantification of TUNEL staining is shown in panel below. (**D**) HC11-TRE and HC11-TRE-CTGF cells were seeded in serum-free media with EGF (10 ng/ml). Lysates were harvested after 96 hours and the expression levels of the indicated survival proteins was analyzed by western blot. Results are representative of 3 experiments.

The apparent ability of CTGF/CCN2 to facilitate integrin-mediated processes and proliferation, suggested a role for CTGF/CCN2 in sustaining the survival of HC11 cells. Therefore, the effect of CTGF/CCN2 expression on the ability of HC11 cells to progress through the cell cycle in the absence of serum was examined. HC11-TRE and HC11-TRE-CTGF cells were harvested following 96 hours in serum-free media, stained with propidium iodide (PI) and analyzed by flow cytometry. Figure 4A contains a cell cycle analysis comparison of the HC11-TRE and HC11-TRE-CTGF cell lines. The data in Figure 4B indicate the percentage of total HC11-TRE and HC11-TRE-CTGF cell populations in the sub G_0_/G_1_, G_1_, S, and G_2_/M phases of the cell cycle. In each cell line the majority of cells were in the G_1 _phase of the cell cycle, however, in support of the data suggesting sustained growth of HC11 cells by CTGF/CCN2, the cells expressing CTGF/CCN2 had greater percentage of the population in both S and G_2_/M phases. In contrast, the HC11-TRE vector control cells showed a greater population of cells in sub G_0_/G_1_, which is indicative of possible apoptosis. To determine the level of apoptosis, the cells were examined for TUNEL, a measure of DNA fragmentation characteristic of cells undergoing apoptosis. The results, shown in Figures 4C, indicated a higher level of TUNEL staining in HC11-TRE cells compared to the HC11-TRE-CTGF cells.

Because CTGF/CCN2 enhanced the survival of HC11 cells, the contribution of CTGF/CCN2 to the activation of integrin-mediated survival signaling was examined. Protein lysates from HC11-TRE-CTGF and HC11-TRE vector control cells were collected after 96 hours in serum-free media and used to determine the expression of mediators of integrin adhesion and survival signaling (Figure 4D). The results indicated that HC11-TRE-CTGF cells display enhanced expression of β1 integrin, phosphorylated FAK, phosphorylated Akt, Bcl-xL and cyclin D1 compared to expression levels in HC11-TRE cells. The level of β3 integrin was slightly increased in the CTGF-expressing cells as well (data not shown). These results indicated that CTGF/CCN2 contributed to integrin-mediated survival signaling in mammary epithelial cells and implicated the β1-containing integrin complex(es) in the control of both proliferation and survival of HC11 cells.

### CTGF/CCN2 increased integrin expression and function in HC11 cells

**Figure 5:**
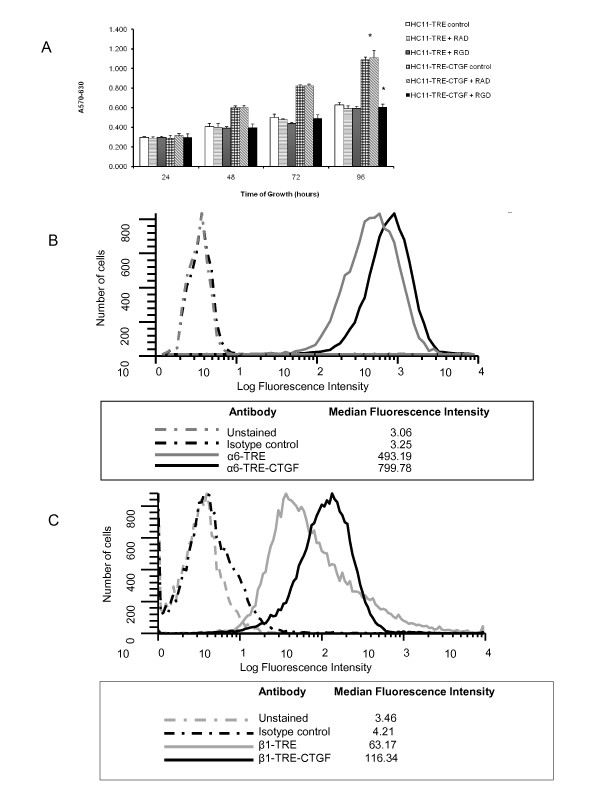
**CTGF/CCN2 contributes to the stabilization of surface-level integrin complexes**. (**A**) HC11-TRE and HC11-TRE-CTGF cells were incubated in serum-free media with EGF (10 ng/ml) or identical media containing either an RGD-containing peptide or RAD-containing peptide (500 μM). The MTT assay was performed at 24, 48, 72, and 96 hours. The results are displayed as the mean + the S.D. of four determinations. *, p < 0.005. (**B**) HC11-TRE and HC11-TRE-CTGF cells were grown in serum free media for 4 days in the presence of EGF (10 ng/ml). Cells were harvested and stained with an anti-β1 integrin antibody, anti-α6 antibody or isotype controls followed by detection with a fluorescence-conjugated secondary antibody and analysis by flow cytometry. The median number of β1 integrin or α6 positive cells is indicated. The data are representative of 4 independent experiments.

CTGF/CCN2 bound to and enhanced the expression of β1 integrin in other cell types [27,46], and the activation of β1 integrin signaling is required for β-casein transcription [47]. Hence, the degree to which the CTGF/CCN2-mediated effects on cell proliferation and survival were dependent on the CTGF/CCN2-induced integrin activation was tested. An MTT assay was performed in the presence of an integrin-binding RGD-containing peptide that would be expected to act as a competitive inhibitor for β1 integrin complexes including αVβ1 and α5β1, as well as αVβ3. HC11-TRE and HC11-TRE-CTGF cells were seeded in serum-free conditions with or without the RGD peptide or control RAD peptide. Levels of proliferation were determined by MTT assay. Results, shown in Figure 5A, displayed the expected enhanced proliferation trend of HC11-TRE-CTGF cells compared to the vector control HC11-TRE cells. The HC11-TRE-CTGF cells incubated with the RGD peptide exhibited a 38% reduction in proliferative capacity at 96 hours, whereas the RAD peptide showed no effect on the CTGF/CCN2-enhanced proliferation of the cells. In contrast, the RGD peptide inhibited growth of the TRE cells by 7% during the same time period. Hence, while the control cells express some endogenous levels of CTGF/CCN2, it is the elevated expression of CTGF/CCN2 that provides a cell adhesion and survival advantage, and targeting those functions exposes the dependence on CTGF/CCN2 in the TRE-CTGF cell line. These results suggested that the RGD peptide partially blocked the integrin engagement that occurred as a result of CTGF/CCN2 expression, thereby affecting growth and survival.

CTGF/CCN2 interacts with the α6β1 integrin complex in other cell types. α6β1 integrin is a common mammary epithelial cell receptor for laminin [17] and β1 is critical for mammary gland development *in vivo *and milk protein synthesis in cultured mammary epithelial cells [20,48]. To evaluate the effect of CTGF/CCN2 on the levels of expression of these integrins, HC11-TRE and HC11-TRE-CTGF cells were incubated with antibodies against surface level α6 or β1 integrin and analyzed by flow cytometry. Results, shown in Figure 5B, indicated that HC11-TRE-CTGF cells displayed a greater level of both α6 and β1 integrin on their surface compared to the HC11-TRE control cells. Together these results suggested that CTGF/CCN2 enhanced proliferation and survival signaling through the stabilization and activation of an α6β1 integrin complex or other α6 and β1 containing complexes.

### HC11 cell adhesion to CTGF/CCN2 required integrin complexes

**Figure 6:**
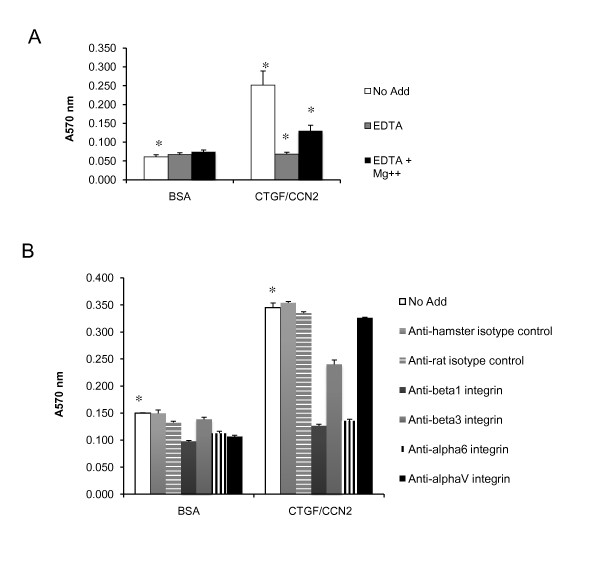
**CTGF/CCN2 enhances adhesion of HC11 cells through the α6β1 integrin complex**. HC11-TRE cells were resuspended in serum-free growth media + EGF (10 ng/ml) and seeded on microtiter wells coated with recombinant CTGF/CCN2 (2 μg/ml) or 1% BSA wells as described in Methods. Adherent cells were fixed , stained, and quantified by reading the absorbance at 570 nm. Data are means of quadruplicate samples, error bars represent standard error. *, ◇, ■, p < 0.005. (**A**) HC11-TRE cells adhesion in serum-free growth media + EGF (10 ng/ml) with EDTA (2.5 mM), EDTA + Mg2+ (5 mM) or no addition. (**B**) HC11-TRE cells adhesion in serum-free growth media + EGF (10 ng/ml) with function-blocking antibodies or isotype controls (25 μg/ml).

CTGF/CCN2 is a secreted, integrin binding molecule. To determine if CTGF/CCN2 was acting to mediate cell adhesion, the ability of HC11 cells to attach to either control or CTGF/CCN2-coated plates was tested. HC11-TRE control cells were allowed to attach to recombinant CTGF/CCN2-coated microtiter wells for 4 hours. Adherent cells were fixed and stained and the absorbance at 570 nm was determined for quantitation of cell adhesion. A significant increase in the adhesion of cells seeded on CTGF/CCN2-coated wells was observed compared with cells seeded on the BSA-coated control wells (Figure 6A). The adhesion was inhibited by the inclusion of EDTA, and rescued with the addition of Mg^2+^, indicative of the interaction required for CTGF/CCN2-integrin-mediated attachments [49].

To determine if the α6β1 integrin complex was involved in the CTGF/CCN2-mediated enhancement of adhesion, the adhesion assay was performed in the presence of function blocking antibodies against both α6 and β1 integrins. CTGF/CCN2 also binds αVβ3 so function blocking antibodies against αv and β3 integrins were tested as well (Figure 6B). The addition of the anti-α6 or anti-β1 antibodies completely blocked the CTGF/CCN2-mediated increase in adhesion while the addition of the anti-β3 showed less inhibition, and the anti-αV or isotype control antibodies showed no significant change in CTGF/CCN2-mediated adhesion. Together with the flow cytometry data demonstrating the CTGF/CCN2-mediated enhancement of surface level expression of the α6β1 integrin complex, these results confirmed the involvement of the α6β1 integrin complex in the CTGF/CCN2-mediated increase in HC11 cell adhesion.

### CTGF/CCN2 expressing cells displayed increased formation of focal adhesions

**Figure 7:**
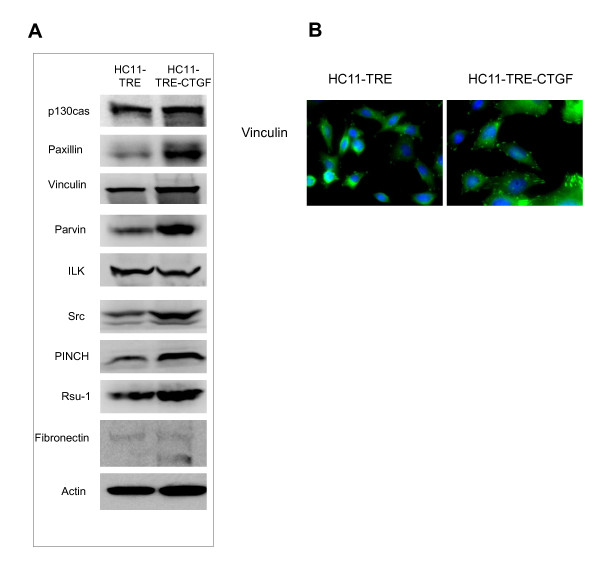
**CTGF/CCN2 contributes to the formation of focal adhesion complexes in HC11 cells**. (**A**) HC11-TRE and HC11-TRE-CTGF cell protein lysates were used to detect levels of the indicated focal adhesion-related proteins by western blot. (**B**) HC11-TRE and HC11-TRE-CTGF cells were seeded in 0.5% serum-containing media with EGF (10 ng/ml) for 4 days. The cells were then fixed in paraformaldehyde and stained for the presence of vinculin with Alexaflor488-conjugated secondary antibody and a DAPI nuclear counterstain. In the vinculin panel focal adhesions are shown as punctate bodies primarily near the edges of cells. Magnification = 40×.

Interactions between mammary epithelial cells and the basement membrane are crucial for survival. To further understand the effects of CTGF/CCN2 elevation and stabilization of β1 integrin complexes on the formation of focal adhesion complexes, the level and the appearance of focal adhesion and extracellular matrix proteins were analyzed. Protein lysates from HC11-TRE and HC11-TRE-CTGF cells were used for determination of specific protein expression by western blot (Figure 7A). Results revealed increases in the focal adhesion-associated adaptor and structural proteins in HC11-TRE-CTGF cells including paxillin, p130CAS, vinculin, parvin, PINCH1, and Rsu-1 [50]. Furthermore, HC11-TRE-CTGF cells displayed increases in Src but not integrin linked kinase (ILK). Elevated transcription of collagen, laminin and fibronectin was detected in HC11 TRE-CTGF cells compared to the TRE control cell line (data not shown) and elevated fibronectin was detected in HC11-TRE-CTGF cells by western blot (Figure 7B). Immunofluorescent staining of the cells with an antibody to vinculin revealed greater focal adhesion formation, both in size and number, in HC11-TRE-CTGF cells compared to vector control HC11-TRE cells (Figure 7B), demonstrating the functionality of CTGF/CCN2 on integrin-dependent formation of focal adhesions in HC11 mammary epithelial cells.

In conclusion, these results indicated that the mechanism by which CTGF/CCN2 contributed to lactogenic differentiation was via activation of integrin-mediated adhesion complex(es), particularly the α6β1 complex, which enhanced cell adhesion and survival in HC11 cells. This could be attributed to direct binding of CTGF/CCN2 to integrin complexes and to CTGF/CCN2-induced laminin and fibronectin expression. Both events would be expected to result in elevated integrin expression and functionality. Because the engagement of β1 integrin is required for the transcription of β-casein and lactogenic differentiation of the mammary gland, these results suggest an important role for CTGF/CCN2 in mammary epithelial cell function.

## Discussion and Conclusions

Stromal mediators play a fundamental role in promoting mammary differentiation, and the contact between mammary epithelial cells and components of the basement membrane contributes to the prolactin-induced transcription of milk proteins [47]. Using the Tet-off system for inducible CTGF/CCN2 expression in the HC11 mouse mammary epithelial cell background, CTGF/CCN2 not only enhanced the early transcription of β-casein in response to lactogenic hormone, but also contributed to the formation of mammospheres, a hallmark of terminal differentiation in these cells. In addition, exogenous CTGF/CCN2 enhanced the formation of differentiated MCF10A acini in three dimensional cultures, extending the role of CTGF/CCN2 to another culture system which is likely to be structurally and physiologically relevant to the *in vivo *setting [42]. The role of CTGF/CCN2 in lactogenic differentiation may depend on its function in the expression and stabilization of matrix-integrin complexes, an activity which promotes prolactin-induced Stat5 activity.

The data reported here suggest that CTGF/CCN2 increased expression of fibronectin and laminin and stabilized the surface expression of the α6 and β1 integrins. Typically, epithelial cells lose their ability to survive in the absence of serum and detach, initiating an apoptotic process called anoikis. However, CTGF/CCN2 served as a survival mediator thereby preventing HC11 cells from undergoing apoptosis in the absence of serum. The binding of integrins to matrix proteins promotes conformational changes in the extracellular domains of the integrins and a subsequent clustering of integrins in focal adhesions. Survival signaling resulting from the formation of focal adhesion complexes involves p130cas, paxillin, ILK, and Src [51]. The complex is formed after adhesion-induced phosphorylation of FAK-Y397. Recruitment of Src causes tyrosine phosphorylation of additional sites on FAK, and FAK-mediated phosphorylation of p130cas and paxillin [52]. FAK activation may also contribute to cell survival by direct activation of the P-I-3 kinase/Akt pathway [53]. CTGF/CCN2 expression correlated with enhanced expression levels of the major proteins involved in the focal adhesions referred to above, as well as PINCH1 and Rsu-1, which localize to focal adhesion complexes [54,55]. Anti-vinculin immunofluorescence visualized larger focal adhesions in CTGF/CCN2-expressing cells compared to control HC11 cells. These results confirm a role for CTGF/CCN2 in the stabilization of active integrin-mediated complexes and adhesion-activated survival signaling. This is likely linked to the CTGF/CCN2-mediated adhesion signaling via integrins and the activation of Akt and expression of Bcl-xL. However, HC11 cells expressing CTGF/CCN2 also exhibited elevated levels of Map kinase phosphatase1 (MKP-1) (data not shown), a dual specificity phosphatase frequently induced in response to Map Kinase signaling. MKP-1 dephosphorylates p38 kinase, suggesting that preventing the accumulation of phospho-p38 may be a contributing mechanism by which CTGF/CCN2 inhibits apoptosis.

CTGF/CCN2-mediated activation of β1 integrin signaling has been demonstrated in primary skin fibroblasts [56] and activated pancreatic stellate cells [46]. In fibroblasts CTGF/CCN2 also induced expression of the extracellular matrix proteins laminin, collagen, fibronectin and integrin subunits [57], similar to the increase in these proteins that was observed in the HC11 cells engineered to express CTGF/CCN2. The elevated expression of fibronectin and laminin would be expected to the increase integrin engagement and focal adhesion formation. Moreover, Cyr61/CCN1 and Nov3/CCN3, highly homologous members of the CCN family, have been shown to bind to and enhance β1 integrin-mediated functions in primary skin fibroblasts [49,58]. In our study enhanced integrin-dependent signaling may have resulted from direct interaction between integrins and secreted or cell-bound CTGF/CCN2, as suggested by the integrin blocking antibody data in Figure 6. In addition, the elevation of matrix protein expression may have increased RGD-binding integrin engagement in HC11 cells and this likely contributed to enhanced proliferation and survival of HC11 TRE-CTGF cells in the absence of serum. Another contribution may result from elevation of syndecan 4, a heparin sulfate proteoglycan, which has been reported to stabilize integrin complexes, including β1 integrin complexes [59]. Syndecan 4 upregulation has been observed in CTGF/CCN2-stimulated or -expressing cells [60,61], and the HC11-TRE-CTGF cells also express elevated levels of syndecan 4 compared to the HC11-TRE vector control cell line (data not shown).

The association of mammary epithelial cells with laminin is critical for the activation and the nuclear translocation of Stat5 [25]. Association of the cells with the matrix proteins occurs through active binding to β1 integrin complexes on the cell surface. It is known that binding to laminin via the β1 integrin complex [3] is directly associated with the induction of lactogenesis and β-casein transcription [5], although the α integrin has not been definitely identified [62,21]. Our data indicate that CTGF/CCN2 enhances the expression of laminin resulting in decreased requirement for exogenous laminin in the activation of β-casein transcription, as seen in Figure 2. CTGF/CCN2 expressing cells did not display the same laminin-dependent increase in the level of β-casein mRNA as control cells, suggesting that CTGF/CCN2 partially abrogates the requirement for those proteins for the transcription of β-casein. Collectively, these results suggest that the mechanism by which CTGF/CCN2 enhances lactogenic differentiation is through enhancement and stabilization of the laminin- α6β1 integrin cell-matrix interaction that is required early in the lactogenic differentiation process.

The adhesion of epithelial cells to extracellular matrix initiates signaling mechanisms that control cell proliferation, survival, and differentiation throughout the phases of mammary gland development. Our finding that ectopic expression of CTGF/CCN2 enhanced the growth and survival of HC11 cells *in vitro *supports a proposed role for CTGF/CCN2 during late pregnancy and early lactation, a period when CTGF/CCN2 levels are elevated *in vivo *[27,28]. While this study focused on the mechanism of CTGF/CCN2 in HC11 mouse mammary epithelial cells, CTGF/CCN2 also enhanced the transcription of β-casein in primary mouse mammary epithelial cells stimulated to differentiate [27]. Recent mammary specific knockdown studies ascertained that β1 integrin as well as FAK and Src contribute to the development of the mammary gland and the induction of lactogenesis [22,63,64]. Mammary specific inhibition of β1 integrin reduced the activation of the MAPK and Akt and ultimately resulted in diminished mammary growth and increased mammary developmental defects [20,63]. Similarly, a mammary specific knockout of FAK caused tissue defects as well as a reduction in milk protein production; this was attributed to a diminished secretory capacity of the epithelium due to the combination of decreased proliferative ability and a lack of Stat5 activation [22]. Moreover, a knockout of Src blocked lactational ability of the mammary gland although the gland maintained otherwise normal growth [64]. Based on the results reported here, the effects of a mammary specific knock down of CTGF/CCN2 may prove useful in understanding the control of lactogenic differentiation *in vivo*.

## Methods

### Cell culture and the construction of CTGF Tet-off cell line

HC11 mouse mammary epithelial cells, provided by Dr. Nancy Hynes, were maintained in RPMI 1640 medium supplemented with 10% fetal bovine serum, 5 μg/ml insulin, 10 mM HEPES and 10 ng/ml EGF as described [13,35]. The HC11 cell line containing the pTet-Off plasmid (Clontech, Mountainview, CA) was infected with a pREV-TRE (Clontech) retroviral vector encoding epitope-tagged CTGF/CCN2 and used to express HA-CTGF/CCN2 in response to removal of doxycycline (Dox) as described previously [27]. The HC11-TRE vector control cells and HC11-TRE-CTGF cells were grown in doxycycline (2 μM) which was removed for 96 hours to induce CTGF/CCN2 expression for experiments.

Prior to stimulation with lactogenic hormones, HC11 cells and HC11-derived cell lines were grown to confluence and maintained for 3-5 days to establish competence [9,39] then maintained in media without EGF for 24 hours. To induce lactogenic hormone-induced differentiation, the cells were rinsed twice and then incubated in differentiation media, RPMI with dexamethasone (10^-6^M), 5 μg/ml insulin, and 10 μg/ml prolactin, referred to as DIP. Lactogenic differentiation was characterized by analysis of β-casein transcription and by the formation of domed structures referred to as mammospheres, which were visualized by phase contrast microscopy and manually enumerated.

### MCF10A acinus formation

Acinus formation was performed with MCF10A cells using an established technique with minor modifications [42]. Growth factor reduced Matrigel was added to wells of an eight-well glass chamber slide. Basement membrane was allowed to solidify for 15 minutes at 37°C. MCF-10A cells were harvested, washed in DMEM/F12, 2% horse serum and resuspended in assay media (DMEM/F12, 2% horse serum, 0.5 μg/ml hydrocortisone, 100 ng/ml cholera toxin, 10 μg/ml insulin). A mixture of assay media containing EGF (20 ng/ml) plus 4% Matrigel was prepared with or without CTGF/CCN2 (25-100 ng/ml, Cell Sciences, Canton, MA). A 1:1 mixture of the Matrigel-containing assay media and MCF-10A cells (25,000 cells/ml) was plated on top of the solidified basement membrane. The cells were incubated at 37°C for 20 days with media changes every 4 days. Each well was fixed with 2% paraformaldehyde followed by permeabilization in 0.5% Triton X-100 at 4°C. Wells were then rinsed with PBS containing glycine (100 mM) and blocked with IF buffer (7.7 mM sodium azide, 0.1% BSA, 0.2% Triton X-100, 0.05% Tween-20, 10% FBS). The cells were washed in PBS and mounted to slides with ProLong Gold antifade reagent containing DAPI (Molecular Probes, Invitrogen, Eugene, OR). Confocal imaging was performed on a Zeiss Pascal Laser Scanning Confocal Microscope (Carl Zeiss, Thornwood, NY).

### RNA isolation, RT-PCR, northern blotting, and Southern blotting

Total RNA was isolated and purified using the TriPure reagent (Roche, Palo Alto, CA). Northern blots were prepared with 10 μg of total RNA separated on a 1% agarose-formaldehyde gel and transferred to a nylon filter. Reverse transcriptase PCR (RT-PCR) was performed using the GeneAmp RNA PCR Kit and primers for β-casein amplification as follows: Fwd 5' CATCCTTTCAGCTTCACC, Rev 5' AGAGACAGCTGGGTCTGAG. Primers for mouse β-actin were as follows: Fwd 5' CTAAGGCCAACCGTGAAAAGA, Rev 5' GAGGTCTTTACGGATGTCAAC. PCR products were electophoresed on a 1% agarose gel. The DNA was denatured and neutralized and transferred to a nylon filter. Northern and Southern blots were hybridized as described previously [27]. The probes for CTGF, β-casein and actin have been described [27,37].

### Chromatin Immunoprecipitation

Confluent HC11-TRE and HC11-TRE-CTGF cells were stimulated with DIP for 16 hours prior to cross-linking with 1% formaldehyde for 10 minutes at room temperature, followed by treatment with 1.12 ml of 2.5 M glycine for 5 minutes at room temperature. The precipitating antibodies used included anti-rabbit IgG (Chemicon, PP64) or anti-Stat5 N-term (Santa Cruz, sc-836). Primers for the β-casein proximal promoter, forward 5'CCAGCTTCTGAATTGCTGCC3', reverse 5' GGTCTATCAGACTCTGTGAC3', were used in PCR reactions of 35 cycles of 1 minute at 95°C, 30 seconds at 55°C, 30 seconds at 72°C. Amplified DNA was analyzed by electrophoresis on an ethidium bromide stained 1.5% agarose gel. Bands were quantitated using the Fujifilm LAS-4000 (Fujifilm Medical Systems USA, Inc.). Results are expressed as a ratio of immunoprecipitated DNA to the input DNA.

### Western blotting and Immunoprecipitation

The procedures for western blotting have been described [27,37,38,55]. The antibodies used in this study include: mouse anti-β-actin (Sigma, St. Louis, MO), goat anti-CTGF (Santa Cruz Biotechnology, Santa Cruz, CA), mouse anti-β1 integrin (BD Transduction, BD Biosciences, San Diego, CA), mouse anti-FAK (BD Transduction), rabbit anti-phospho-FAK (Upstate Technologies), rabbit anti-Akt (Cell Signaling Technolgies, Danvers, MA ), rabbit anti-phospho-Akt (Cell Signaling Technology), rabbit anti-Bcl-xL (Santa Cruz Biotechnology), mouse anti-PINCH1 (BD Transduction), rabbit anti-ILK (Santa Cruz Biotechnology), rabbit anti-actopaxin/parvin (Sigma), mouse anti-p130CAS (Upstate Technologies), mouse anti-paxillin (BD Transduction), rabbit anti-c-src (Santa Cruz Biotechnology), mouse anti-vinculin (Sigma). The rabbit anti-Rsu-1 antibody has been described [55].

### Immunofluorescence

To detect focal adhesions, HC11-TRE and HC11-TRE-CTGF cells were grown in the absence of doxycycline for 4 days, at which time they were seeded on coverslips and maintained in serum-free media + EGF (10 ng/ml) for 4 days. The cells were washed with PBS, fixed in 4% paraformaldehyde then permeabilized with 0.5% Triton X-100 and blocked in 4% BSA prior to an overnight incubation at 4°C with an anti-vinculin antibody (Sigma). Coverslips were washed with PBS and incubated for 30 minutes in the dark at room temperature with an AlexaFluor 488-conjugated secondary antibody then mounted with ProLong Gold antifade reagent containing DAPI (Molecular Probes, Invitrogen, Eugene, OR). Immunofluorescence was visualized using an Olympus IX71 microscope, a QImaging Retiga 2000RV camera, and QCapture Pro 6.0 software (Olympus America Inc, Center Valley, PA).

### MTT and TUNEL Assays

Proliferation of HC11-TRE-CTGF and vector control HC11-TRE cells was determined by MTT assay (CellTiter96 Assay, Promega, Madison, WI). Viable cells grown in the absence of doxycycline for 96 hours were replated at a density of 1.5 × 10^4 ^per well in quadruplicate wells of a 96-well plate in serum-free media with EGF (10 ng/ml). HC11-TRE cells were analyzed following exposure to conditioned media from HC11-TRE-CTGF cells, and in the presence of RGD- or RAD-containing peptide (500 μM, BioMol, Plymouth Meeting, PA). The cells were incubated for 24, 48, 72, or 96 hours. Analysis of the MTT assay has been described previously [37].

Apoptosis was detected in vector control HC11-TRE cells and HC11-TRE-CTGF cells by TUNEL technology (*In Situ *Cell Death Detection Kit, Fluorescein, Roche). Cells previously grown in the absence of doxycycline for 96 hours were grown on coverslips in serum-free media in the presence of EGF (10 ng/ml) for 96 hours. The cells were fixed in 4% paraformaldehyde, permeabilized in 0.1% Triton X-100 on ice, and rinsed in PBS and incubated in 50 μl TUNEL reagent for 1 hour at 37°C in the dark. The cells were rinsed in PBS prior to being mounted on slides (Vectashield mounting media, Vector Labs, Burlingame, CA). Cells were viewed on the Olympus BX61 and analyzed by IVision software (IVision, Atlanta, GA).

### Flow cytometry

Cell cycle analysis and surface level expression of α6 and β1 integrins were determined by flow cytometry. HC11-TRE and HC11-TRE-CTGF cells grown without doxycycline for 96 hours were propagated for 96 hours in serum-free media with EGF (10 ng/ml). The cells were harvested with Cell Stripper (Mediatech, Manassas, VA), pelleted, washed, and counted. For cell cycle analysis, cells were resuspended in PBS and methanol was added dropwise for fixation prior to storage at -20°C for 24-96 hours. The cells were pelleted and resuspended in cold PBS and propidium iodide (50 μg/ml) was added to the cells prior to flow cytometric analysis. For integrin expression analysis, the cells were aliquoted into 1 ml samples of 500,000 cells each and washed in FACS wash buffer (1% FBS, 0.05% sodium azide). Cells were then incubated in buffer with antibodies against β1 integrin (BD Biosciences) or α6 integrin (BD Biosciences) or the isotype controls for 1 hour at 4°C then pelleted and resuspended FACS buffer containing PE- or FITC- conjugated secondary antibodies for 45 minutes at 4°C. The cells were fixed in Cytofix (BD Biosciences) on ice, washed and resuspended in FACS buffer. All flow cytometry was performed using a BD Biosciences LSRII cytometer. Cell cycle analysis was performed using ModFit LT software and integrin expression analysis was performed using WinList software (Verity Software House Inc.).

### CTGF/CCN2 adhesion assay

To determine the interaction between CTGF/CCN2 and integrin complexes, HC11-TRE cells were seeded in serum-free media + EGF (10 ng/ml) on maleic anhydride Reacti-Bind microtiter wells (Thermo Scientific, Rockville, MD) coated with recombinant CCN2 protein (Cell Sciences, Canton, MA). The plates were coated with CTGF/CCN2 (2 μg/ml) overnight at 4°C, followed by blocking with 1% BSA for 2 hours at 37°C. HC11-TRE cells were suspended at a concentration of 5 × 10^5 ^cells/ml, and where indicated mixed with EDTA (2.5 mM), EDTA (2.5 mM) + Mg^2+^, blocking antibodies to αV, β3 (Santa Cruz Biotechnologies), α6, or β1 integrins (BD Biosciences), or isotype control antibodies. Cells were allowed to attach for 4 hours at 37°C, washed gently with PBS and fixed with 3.7% formaldehyde for 10 minutes at room temperature. Fixed cells were washed with stained with crystal violet and the adherent cells were quantified by reading the absorbance at 570 nm.

## Abbreviations

CTGF: Connective Tissue Growth Factor; FAK: Focal Adhesion Kinase; RGD: Arginine-Glycine-Aspartic Acid; RAD: Arginine-Alanine-Aspartic Acid

## Competing interests

The authors declare that they have no competing interests and that the funding agencies did not influence the design of the project or the preparation of the manuscript.

## Authors' contributions

BM performed differentiation experiments, expression studies, binding assays, immunofluorescence and flow cytometry and drafted the manuscript. CJ performed some western blots, RNA isolation and northern blots. MLC conceived of the project, participated in its design and coordination and prepared the final draft of the manuscript. All authors read and approved the final manuscript.
